# Renal Lymphoma Mimicking a Retroperitoneal Hematoma

**DOI:** 10.7759/cureus.15099

**Published:** 2021-05-18

**Authors:** Ana Primitivo, Pedro M Sousa, Ana F Ferreira

**Affiliations:** 1 Radiology, Hospital Beatriz Ângelo, Loures, PRT

**Keywords:** primary renal lymphoma, lymphoma, non-hodgkin, kidney

## Abstract

We report the case of a 65-year-old female with an atypical presentation of renal lymphoma at computed tomography (CT), which was initially misinterpreted as a retroperitoneal hematoma. This case highlights the importance to keep a high level of suspicion in order to make a prompt diagnosis since treatment strategies differ significantly.

## Introduction

Lymphoma is the most common malignant retroperitoneal tumor. Compared with lymphoma in other locations, retroperitoneal lymphoma depicts more prone to form confluent soft-tissue masses. In such cases, lymphoma may closely resemble other possible benign entities such as retroperitoneal fibrosis and hematoma leading to possible misdiagnosis.

In this case report, we present a patient which was initially misdiagnosed with retroperitoneal hematoma, and we discuss some imaging pearls that may aid in the differential diagnosis.

## Case presentation

**Figure 1:**
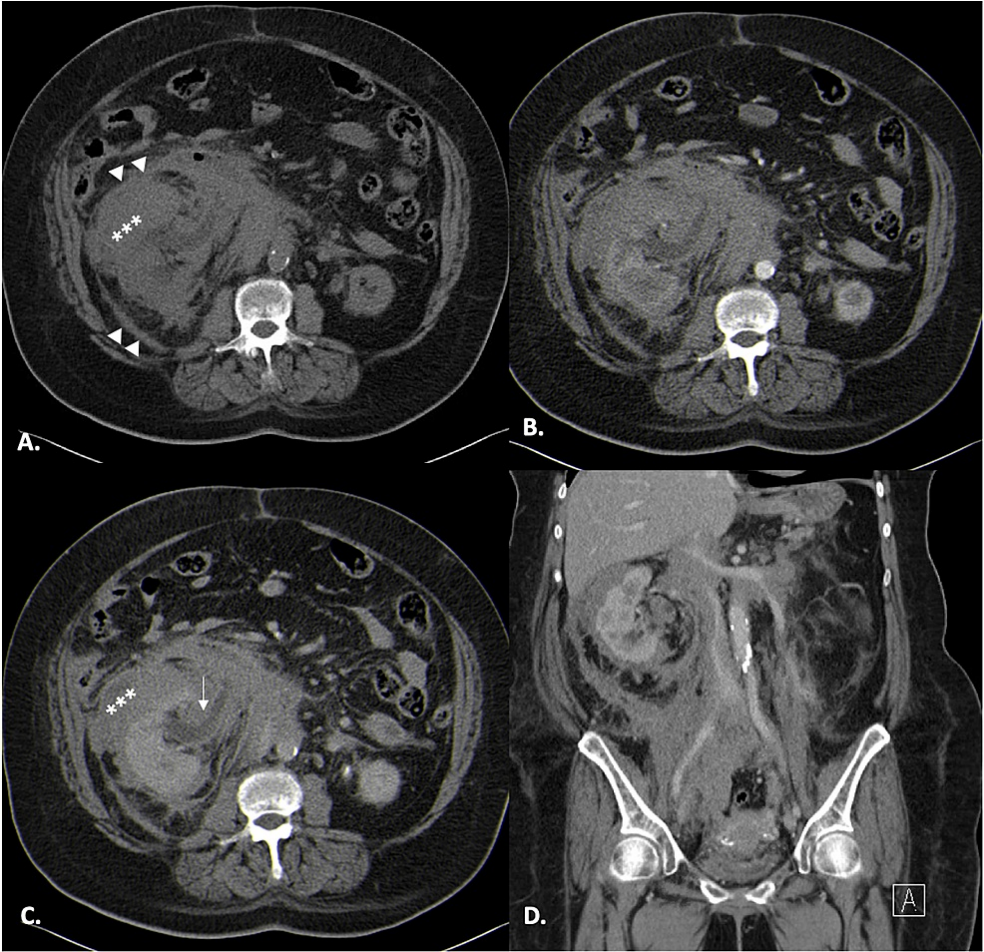
Initial CT scan of a 65-year-old female with primary renal lymphoma. Axial non-contrast CT (A) image of the right kidney depicts densification of the perirenal and anterior pararenal right spaces with mean of 35 HU (white asterisks in A and C); thickening of the anterior and posterior renal fasciae (white arrowheads in A). Axial arterial and portal phases (B and C) at the same level show slight enhancement, although less than the renal cortex (with mean HU of this densification in the arterial phase of 47 and in the portal phase 65). Coronal imaging at portal venous phase (D) depicts the vessels slightly molded but not invaded, and still permeable. Also, note associated right hydronephrosis (white arrow in C).

A 65-year-old female presented to a routine medical appointment for her diabetic nephropathy, with cough and dyspnea for one week without fever. Medications taken at that time included losartan 100 mg, lercanidipine 20mg, furosemide 40 mg, insulin 100 UI/mL, warfarin 5 mg, and atorvastatin 40 mg. The initial white blood cell count was 11.41 (normal range of [4.0-10] x 10/L), prothrombin time 80 (10.6-13.5s), international normalized ratio (INR) 9.33, amylase 95 (28-100 U/L), lipase 190 (13-69 U/L), lactate dehydrogenase (LDH) 838 (< 248 U/dL), C-reactive protein (RCP) 7.53 (<0.50 mg/dl) and creatinine 3.80 (0.5-0.90 mg/dl). A computed tomography (CT) was then performed for further evaluation. Axial contrast-enhanced CT showed an extensive retroperitoneal lesion centered at the right perirenal space and extending anteriorly to the anterior pararenal space. The lesion demonstrated hyperdense areas with a mean of 35 Hounsfield units (HU) at nonenhanced CT (Figure 1 A). After contrast, this densification enhanced, although slightly less than the renal cortex (Figure 1B and C).

 

This lesion involved circumferentially the abdominal vessels and the right ureter causing hydronephrosis. This was initially thought to be a spontaneous retroperitoneal hematoma, taking into consideration the abnormal coagulation values. Although other differentials were considered, such as lymphoma and retroperitoneal fibrosis. The patient did a follow-up CT one month later, to evaluate the anticipated interval resolution of the hematoma. 

**Figure 2:**
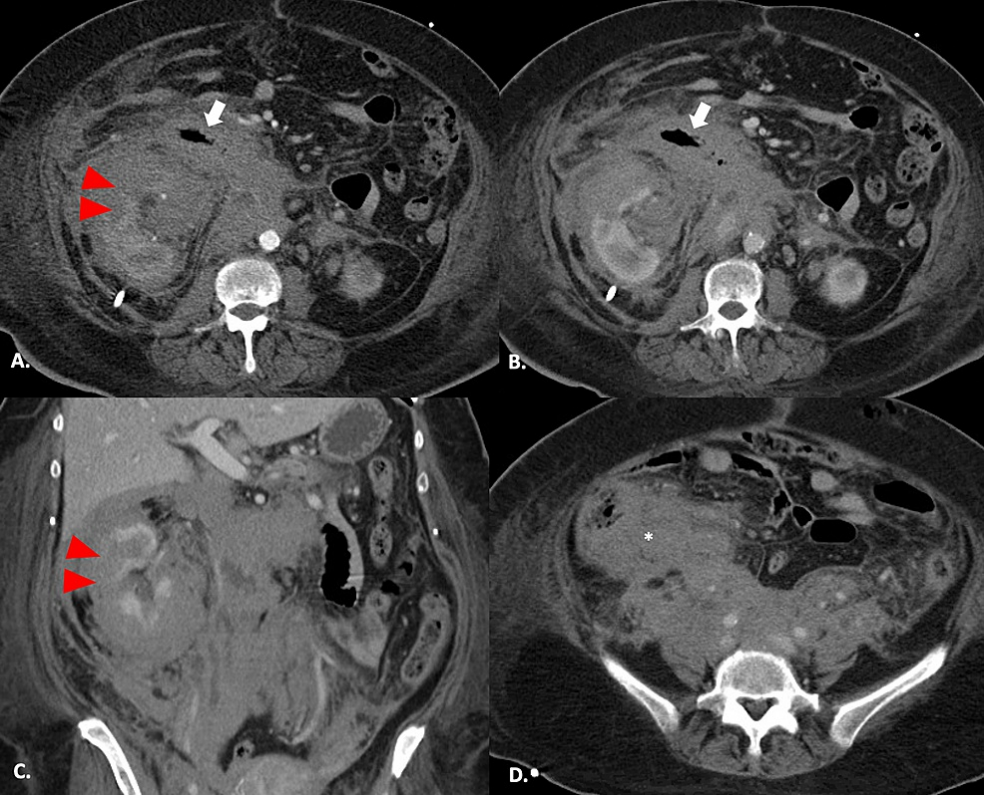
Follow-up CT scan acquired one month after the initial presentation. Axial arterial and portal enhanced CT images (A and B) demonstrate an increase of its extension. The densification now involves circumferentially the duodenum (white arrows) reducing its lumen. Portal venous phase (C) depicts markedly irregularity of the right kidney (red arrowheads) and encasement of the renal vessels. Axial portal venous phase (D) showing peri-cecum densification with loss of the fat cleavage plan (white asterisk). There wasn't significant variability of attenuation compared to the initial evaluation. These findings ruled out the hypothesis of hematoma, and strongly suggest a lymphoproliferative disorder.

Instead follow-up CT demonstrated an increase in its extension, with the invasion of the duodenum, reducing its lumen (Figure 2A and B). Of note, there was now counter irregularity of the right kidney and extension to renal parenchyma (Figure 2B and C). There was also peri-cecum densification with loss of the normal fat cleavage plane (Figure 2D). There was no significant variability of density compared to the initial evaluation. These overall findings and the interval growth strongly suggested a lymphoproliferative disorder. 

A percutaneous guided CT biopsy was performed and the final diagnosis was a diffuse large B-cell lymphoma. Immunohistochemical analyses demonstrated positivity for CD20 and negativity for CD3 and AE1/AE3.

## Discussion

Lymphoma may be unifocal, multifocal, or diffuse. It may affect isolated lymph nodes or any organ system, and demonstrate a wide range of imaging appearances at almost every site [1]. Extranodal lymphoma includes renal lymphoma, which may occur isolated or more commonly as part of multiple organ involvement [2,3].

Primary renal lymphoma accounts for <5% of all cases of renal lymphoma. Imaging features may include diffuse enlargement or focal renal masses. Circumferential perirenal soft tissue and direct renal extension are usually detected at the onset [3].

The majority of cases of renal lymphoma are clinically asymptomatic and incidentally founded at imaging. Immunocompromised patients are at significantly higher risk for developing lymphoma [4].

CT remains the main imaging modality for initial diagnosis, staging, and monitoring of the disease, as not only depicts the renal lesions but also helps to identify extension to adjacent anatomic structures such as the perirenal space and the retroperitoneum, thus helping to determine the systemic spread of the disease [3,5].

The role of ultrasound (US) is limited, but may be useful as a guidance modality for percutaneous biopsy. The lesions are usually hypoechoic compared to normal renal parenchyma [6].

On CT it features as a soft-tissue homogenous mass, slightly hyperdense compared to the adjacent unenhanced renal parenchyma [4]. On MRI, lymphoma demonstrates hypointense signal on T1-weighted images (WI) and is slightly hypointense or isointense relative to normal renal cortex on T2-WI. After contrast, lymphomatous deposits enhance less than the surrounding normal parenchyma in both CT and MRI. However, some lesions may show late progressive enhancement [7].

Lymphomas may appear as large masses, as in our case, invading or displacing the adjacent kidney. Entrapment of the right ureter caused hydronephrosis, which is commonly seen in lymphomas.

Thrombosis of major renal arteries and veins is rare despite extensive tumor encasement. It may also appear with less striking features, namely limited thickening of the peri-renal fasciae or plaques and nodules in the perirenal space [8].

At FDG PET/CT, renal involvement in NHL appears as multiple focal areas of increased uptake in the renal cortex [7].

The differential diagnosis includes sarcoma arising from the renal capsule, metastatic tumor, as well as benign conditions such as perinephric and retroperitoneal hematoma, retroperitoneal fibrosis (RPF), retroperitoneal tuberculosis, amyloidosis, and extramedullary hematopoiesis [3].

Hematomas are avascular with possible hematocrit-fluid levels. Subcapsular fluid collection typically demonstrates density around 30-50 Hounsfield Units (HU). A sentinel clot or active extravasation may be seen. Non-enhancing subcapsular masses with variable densities, depending upon the age of blood products are also common [4,9].

Regarding retroperitoneal fibrosis (RPF) it may be indistinguishable from lymphoma, specifically in terms of enhancement pattern. RPF is usually located anteriorly or laterally to the aorta and unlike lymphomas, the aorta is usually not displaced forward. The attenuation tends to be more homogeneous. Lymphoma may have areas of necrosis, with greater lesions and associated lymph nodes [10].

Another possible differential diagnosis is retroperitoneal tuberculosis. However, there were not rim enhancement either enlarged lymph nodes which would have favored this hypothesis [11].

Core biopsy, flow cytometric, and immunohistochemical studies are very important in confirming the diagnosis and classifying the tumor subtype prior to therapy.

Immunohistochemical stains commonly used include CD20 (B-cell marker), CD3 (T-cell marker), kappa and lambda light chains (which depicts clonality in B lymphocytes), CD15, and CD30 (which were Reed-Sternberg cell markers) [12,13].

Treatment depends on the clinical stage and subtype. It usually encompasses a chemotherapy-immunotherapy or combined-modality therapy [1,5].

The overall prognosis is poor. Median survival times ranging from eight months to three years, and a 40%-50% five-year survival rate [4].

In the subsequent days, her clinical status aggravated significantly, resulting in tumor lysis syndrome and death.

## Conclusions

The kidney is almost always a secondary location for lymphoma. Early diagnosis is crucial because unlike other retroperitoneal tumors surgery does not play a role in the management of lymphoma. The main differential diagnosis may include benign entities such as hematoma or retroperitoneal fibrosis, where short interval follow-up imaging or other imaging modalities may be needed in order to exclude malignancy. A biopsy is mandatory in order to confirm the diagnosis.
